# What is not measured cannot be improved: the urgency to understand causes of HIV‐related deaths in Latin America

**DOI:** 10.1002/jia2.70065

**Published:** 2025-11-27

**Authors:** Blanca Lopez‐Villalba, Monica Alonso‐Gonzalez, Bernardo Nuche‐Berenguer, J. Javier Castrodeza‐Sanz, Omar Sued

**Affiliations:** ^1^ Department of Preventive Medicine and Public Health University Clinical Hospital of Valladolid Valladolid Spain; ^2^ HIV Unit, Communicable Diseases Prevention, Control and Elimination Department Pan American Health Organization Washington DC USA

1

The collection and analysis of HIV‐related mortality trends and their causes in Latin America (LA) are limited, which impedes planning and prioritization of interventions. National HIV surveillance databases within the region typically do not track deaths among people living with HIV directly. Instead, countries report information received from the vital registration (VR) system or rely on modelled statistical estimates. VR system uses the International Classification of Diseases (ICD) to standardize, record and categorize causes of death. While this coding system is essential for monitoring mortality trends, its implementation is often delayed and affected by multiple factors that compromise the quality of death certification [1].

In the case of HIV, the ICD codes B20−24 are used to classify deaths related to conditions listed as WHO Stage 4 or CDC Stage C. However, misclassifications leading to underreporting are common. These often result from the use of ill‐defined codes (i.e. symptoms, signs, and abnormal clinical and laboratory findings such as codes R00−R99) or garbage codes (e.g. listing only the immediate or intermediate causes of death without identifying the underlying cause, often due to lack of knowledge, stigma or confidentiality concerns). Misclassification can also lead to overreporting by incorrectly attributing HIV deaths to diseases that mimic HIV infection or by assigning HIV as the underlying cause in deaths primarily due to other conditions. While globally the misclassification has declined, researchers estimated that between 8.5% and 39.7% of total HIV deaths remain misclassified [2].

In LA, only two studies—over a decade old—have examined how HIV‐related deaths are captured in VR systems. These studies, conducted in Brazil [3] and Mexico [4], highlight the limited availability of data on this topic. Notably, they also identified inaccuracies in death attribution with estimated misclassification rates of around 27% and 11%, respectively. These findings suggest persistent weaknesses in how HIV deaths are coded in LA information systems.

Frameworks to improve the accuracy and comparability of cause‐of‐death data have been developed to estimate trends in HIV mortality. These frameworks account for variability in the completeness of VR data, redistribute deaths attributed to garbage codes and correct for misclassifications [2]. However, their effectiveness is limited by countries’ analytic capacity to conduct such analyses.

One such approach is the Coding Causes of Death in HIV (CoDe) Project, developed by the EuroCoord/EACS collaboration [5]. CoDe uses data collection from multiple sources, such as medical records, autopsy reports and death certificates, and uses a centralized adjudication process with independent reviewers to accurately attribute the cause of death (e.g. AIDS‐related, non‐AIDS‐related, unknown). This methodology has been implemented in major cohort studies, including D:A:D and EuroSIDA [6]. A recent expert consensus—developed by clinicians, public health specialists and researchers—goes beyond cause‐of‐death attribution by focusing on the preventability of HIV deaths to enhance public health actionability [7].

Some of these approaches could be implemented in LA to better understand which HIV deaths can be prevented if the region can address the limitations in the availability, quality and analysis of national HIV mortality data. Improving data collection in accordance with ICD standards is a critical step, which should be supported by enhanced infrastructure, qualified human resources and training for personnel to ensure timely and accurate mortality records.

Establishing clear national procedures to identify and correct inconsistencies in death certificates by enhancing interoperability and automating data integration between HIV surveillance databases and other data sources—such as medical records and laboratory systems—can reduce manual data entry errors and misclassification and enable timely updates. These improvements are especially relevant in LA, where fragmented information systems and limited digital infrastructure hinder the accurate monitoring of HIV‐related mortality. Strengthening these areas would improve data quality and availability, support more precise identification of trends, and ultimately guide evidence‐based public health strategies to prevent HIV‐related deaths.

Several LA countries—Brazil, Mexico, Ecuador, Peru, Colombia, Haiti, Argentina and Uruguay—are making progress in integrating surveillance systems, which enable the linkage of clinical, laboratory and mortality data through unique identifiers, thereby improving longitudinal patient follow‐up. These countries are also prioritizing the integration with other health information systems and the monitoring of HIV outcomes, including advanced disease, opportunistic infections and mortality, although challenges persist [8, 9].

Since August 2024, PAHO, with support from Unitaid, has been assisting countries in LA to reduce HIV‐related mortality through a multi‐component initiative. Of relevance to mortality surveillance, the project includes strengthening national HIV information systems to improve the measurement of advanced HIV and preventable HIV‐related deaths. PAHO is providing direct support to monitor advanced HIV, opportunistic infections and deaths and supporting HIV cause of death studies in the region as part of a large project focused on reducing HIV preventable deaths [10].

A generic protocol has been developed to enable the linkage of multiple data sources—VR, clinical, laboratory and HIV surveillance data—to more accurately determine the true number of HIV‐related deaths, their underlying causes, as well as to analyse the changes that can be implemented in the health system to address them. Guatemala, Paraguay, Cuba and Bolivia initiated these studies at the national level, while National AIDS Programs from Brazil, Uruguay and Honduras have received support from PAHO for improving routine data collection on opportunistic infections and deaths. Additional project activities include implementation research on advanced HIV disease to assess the prevalence and early mortality associated with opportunistic infections. Documented 30‐day mortality ranged between 6.5% in Trinidad and Tobago [11], 10% in Paraguay [12], 13% in Brazil [13], with rates twice as high among individuals with histoplasmosis or cryptococcosis. These infections were the strongest predictors of mortality [14].

Improving national information systems to quantify and analyse causes of death is critical for monitoring the HIV epidemic. PAHO calls for a renovated leadership and commitment of national and local stakeholders to strengthen data systems for capturing advanced HIV disease, opportunistic infections and HIV‐related deaths, as this is essential to enhance the understanding of the epidemic and guide effective interventions to reduce HIV mortality in LA countries towards the ambitious objective to eliminate HIV as a public health threat.

## COMPETING INTERESTS

The authors report no conflicts of interest.

## AUTHORS’ CONTRIBUTIONS

All authors contributed to the writing and approval of the manuscript.

## Data Availability

The data that support the findings of this study are available on request from the corresponding author. The data are not publicly available due to privacy or ethical restrictions.
