# Correction to “What is not measured cannot be improved: the urgency to understand causes of HIV‐related deaths in Latin America”

**DOI:** 10.1002/jia2.70092

**Published:** 2026-03-05

Lopez‐Villalba, B., Alonso‐Gonzalez, M., Nuche‐Berenguer, B., Castrodeza‐Sanz, J.J. and Sued, O. (2025), What is not measured cannot be improved: the urgency to understand causes of HIV‐related deaths in Latin America. *J Int AIDS Soc*., 28: e70065. https://doi.org/10.1002/jia2.70065

In the article, there is a correction in the acknowledgements.

The statement reads:

**ACKNOWLEDGEMENTS**

The authors thank the organizations that contribute to improving the quality of life for people living with HIV and those who conduct research to prevent these deaths and the spread of the disease.

This should read:

**ACKNOWLEDGEMENTS**

The authors thank Unitaid for the support of Zero AIDS Deaths by 2030 PAHO project and other organizations that contribute to improving the quality of life for people living with HIV and those who conduct research to prevent these deaths and the spread of the disease.

